# Guidance on Selecting Optimal Steady-State Tacrolimus Concentrations for Continuous IV Perfusion: Insights from Physiologically Based Pharmacokinetic Modeling

**DOI:** 10.3390/ph17081047

**Published:** 2024-08-08

**Authors:** Romain Martischang, Argyro Nikolaou, Youssef Daali, Caroline Flora Samer, Jean Terrier

**Affiliations:** 1 Division of General Internal Medicine, Geneva University Hospitals, 1205 Geneva, Switzerland; 2 Division of Clinical Pharmacology and Toxicology, Department of Anesthesiology, Pharmacology, Intensive Care and Emergency Medicine, Geneva University Hospitals, 1205 Geneva, Switzerland; 3 School of Pharmaceutical Sciences, Institute of Pharmaceutical Sciences of Western Switzerland, University of Geneva, 1205 Geneva, Switzerland; 4 Faculty of Medicine, University of Geneva, 1205 Geneva, Switzerland

**Keywords:** physiologically based pharmacokinetic modeling, tacrolimus, dosing, precision medicine, therapeutic drug monitoring

## Abstract

**Introduction**: The dose–response relationships of tacrolimus have been primarily assessed through trough concentrations during intermittent administrations. In scenarios where oral administration (PO) is unfeasible, continuous intravenous (IV) administration is advised. Under these circumstances, only steady-state (Css) plasma or blood concentrations are measured, with the absence of distinct trough levels (Cmin). Consequently, the measured concentrations are frequently misinterpreted as trough concentrations, potentially resulting in sub-therapeutic true tacrolimus blood levels. This study employs physiologically based pharmacokinetic modeling (PBPK) to establish the Css/Cmin ratio for tacrolimus across various clinical scenarios. **Method**: Using a validated PBPK model, the tacrolimus dose (both PO and IV) and the Css/Cmin ratios corresponding to matching area under the blood concentration–time curve during a dosage interval (AUCτ) values were estimated under different conditions, including healthy subjects and individuals exhibiting cytochrome P450 3A (CYP3A) interactions or CYP3A5 polymorphisms, along with a demonstration of a real-life clinical application. **Result**: In healthy volunteers, the oral/intravenous (PO/IV) dose ratio was found to be 4.25, and the Css/Cmin ratio was 1.40. A specific clinical case substantiated the practical applicability of the Css/Cmin ratio as simulated by PBPK, demonstrating no immediate clinical complications related to the transplant. When considering liver donors versus recipients expressing CYP3A5, the tacrolimus AUCτ was notably affected, yielding a PO/IV dose ratio of 4.00 and a Css/Cmin ratio of 1.75. Furthermore, the concomitant administration of the CYP3A inhibitor itraconazole given PO resulted in a PO/IV ratio of 1.75 with and a Css/Cmin ratio of 1.28. Notably, the inhibitory effect of itraconazole was diminished when administered IV. **Conclusions**: Through the application of PBPK methodologies, this study estimates the PO/IV dose ratios and Css/Cmin ratios that can enhance dose adjustment and therapeutic drug monitoring during the switch between IV and PO administration of tacrolimus in transplant patients, ultimately guiding clinicians in real-time decision-making. Further validation with in vivo data is recommended to support these findings.

## 1. Introduction

Tacrolimus pharmacokinetics are influenced by a variety of transplant-related and patient-specific factors. It exhibits significant binding to erythrocytes and plasma proteins and is metabolized by hepatic and intestinal cytochrome P450 3A (CYP3A) CYP3A4 and CYP3A5 [1]. Its metabolic clearance is thus further modulated by the graft function, drug interactions, and genetic polymorphisms present in both the donor and the recipient [2,3,4]. Given the narrow therapeutic index of tacrolimus and its heterogeneous pharmacokinetics, therapeutic drug monitoring is essential to ensure optimal dosing and efficacy [5].

The therapeutic concentration targets for tacrolimus in solid transplants vary between institutions and protocols, based on trough concentrations [6,7,8]. The dose–response relationships for tacrolimus are generally evaluated using trough concentrations for intermittent administrations. In scenarios such as liver transplants, where oral administration is often not feasible initially, continuous intravenous administration is recommended at doses of 0.01–0.05 mg/kg/24 h [9]. In this context, only steady-state plasma or blood concentrations (Css) are measured, with no distinct trough levels (Cmin). Currently, there are no established guidelines for optimizing tacrolimus dosing based on steady-state concentrations. As a result, measured concentrations are thus often misinterpreted as trough concentrations, leading to subtherapeutic true tacrolimus blood levels. Using a realistic case from our clinical practice, the Css/Cmin ratios and the resulting oral (PO)/intravenous (IV) dose ratios were defined by integrating the best available evidence from the literature and employing them using physiologically based pharmacokinetic (PBPK) simulations.

A PBPK model integrates a physiological model, based on human anatomy and physiology, with a drug model, which encompasses the drug’s pharmacokinetic properties—absorption, distribution, metabolism, excretion [10]. The mathematical framework is then populated with available in vitro or in silico data. This aims to simulate the drug’s pharmacokinetic profile in plasma or tissues as a function of various intrinsic and extrinsic factors. Such simulations have multiple implications, including informing dosage regimens according to specific drug or patient characteristics [10]. Several PBPK-based models for tacrolimus have been developed and successfully verified for cardiac [11], lung [12], renal and deceased- and living-donor liver transplant patients [4,13], as well as for specific populations [14,15]. However, none of these studies have addressed the question of which Css should be targeted during continuous IV administration of tacrolimus.

Taking advantage of PBPK modeling, various clinical scenarios incorporating CYP3A inhibition and CYP3A5 genetic polymorphism in both donors and recipients were considered to study their impact on the Css/Cmin ratio (the general workflow of the study can be found in Figure S1). The proposed ratios presented here could then guide clinicians in adjusting tacrolimus doses for patients with complex clinical scenarios commonly encountered in real life.

## 2. Results

### 2.1. Clinical Case

A 70-year-old Caucasian female patient with a history of distal gastrectomy with loop-type gastrojejunostomy (Billroth II operation), cirrhosis following treatment for hepatitis C, and multiple recurrences of hepatocellular carcinoma despite multiple thermoablations (2020 and 2021) underwent orthotopic liver transplantation on 14 April 2023. Her immunosuppressive regimen consisted of mycophenolate mofetil, tacrolimus, basiliximab, and corticosteroids. Fourteen days post-transplantation, she developed punctate gastric and duodenal perforations, which were sutured. Due to persistent gastric leakage, a gradual resumption of treatment via nasogastric tube (NGT) was initiated and intravenous immunosuppression with tacrolimus was resumed. Three months after transplantation, the patient experienced a new gastric perforation, complicated by a purulent leakage and severe bleeding. Despite medical intervention, the patient eventually succumbed to hemorrhagic shock. Tacrolimus blood levels were measured at trough levels using the immunoassay (ECLIA Roche^®^ method). Notably, she was not receiving any concomitant CYP3A inhibitory drug, and the CYP3A5 expression status of both the liver donor and the patient was unknown. Her hematocrit was 23.7%.

### 2.2. PBPK Simulation of Tacrolimus after IV Infusion and PO Administration in Healthy Volunteers and Application to the Clinical Case Using Virtal Twining

**Figure 1:**
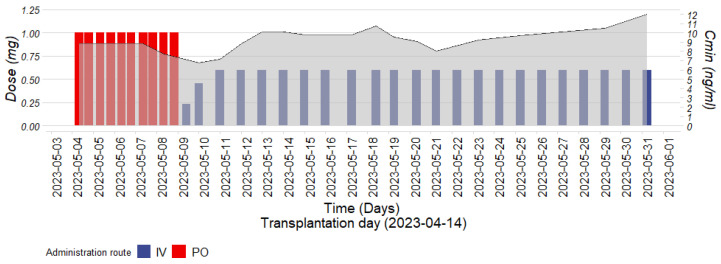
Patient’s tacrolimus trough concentrations (Cmin) (gray), alongside the corresponding oral (PO) doses (red) and intravenous (IV) doses (blue) over time.

After liver transplantation, the patient was prescribed oral tacrolimus (Prograf^®^) capsules at a dose of 1 mg every 12 h, achieving trough concentrations between 8.5 and 9.2 ng/mL within the target range of 8–12 ng/mL (Figure 1). Upon switching IV tacrolimus at a dose of 0.25 mg per 24 h, the clinical pharmacologist recommended targeting a Css/Cmin ratio of 1.4 based on the literature and PBPK simulations. Simulated pharmacokinetic profiles for PO and IV tacrolimus administration in healthy volunteers predicted a Css/Cmin ratio and PO/IV dose ratio of 1.4 and 4.25, respectively, for the same area under the blood concentration–time curve during a dosage interval (AUCτ) (Table 1). Consequently, the IV tacrolimus (Prograf^®^ injection) dosage was increased to 0.6 mg per 24 h. Despite expected intra-individual variations in a critically ill patient, steady-state concentrations ranged from 9 to 12 ng/mL (a Css/Cmin between 0.98 and 1.41 with a PO/IV dose ratio of 3.33) (Figure 1). Until the patient’s death, no signs of graft rejection were observed. We also retrospectively simulated the patient’s pharmacokinetic profile using a virtual twin approach, incorporating her demographic characteristics [16]. Both the donor and the patient were considered non-CYP3A5 expressors due to their Caucasian ethnicity. Their individual Css/Cmin and PO/IV ratios were slightly lower (1.25 vs. 1.40 and 3.92 vs. 4.25, respectively) compared to population predictions, which includes a small percentage of CYP3A5 expressors (17%) (see Section 4. Material and Methods), and were closer to the observed values (Table 1). For the individual simulation, liver function was considered normal since the graft was 1 month old and regular follow-up indicated normal graft perfusion [2,17]. We also performed an analysis considering a decline in liver function, as might occur in the early post-transplantation period (Figure S1). A decrease in CYP3A4/5 liver abundance slightly reduced the Css/Cmin, which remained stable at 1.16–17, and slightly decreased the PO/IV ratio to 3.77 for a 90% reduction in liver function. Since a normally functioning graft was considered for the simulation, all subsequent PBPK simulations were performed in a healthy volunteer population (see Section 4. Material and Methods).

### 2.3. Predicted Pharmacokinetic Parameters of Tacrolimus after PO Administration and IV Infusion in Donors, Recipients and in Both Donors and Recipients Expressing CYP3A5 (1*/1*)

**Figure 2:**
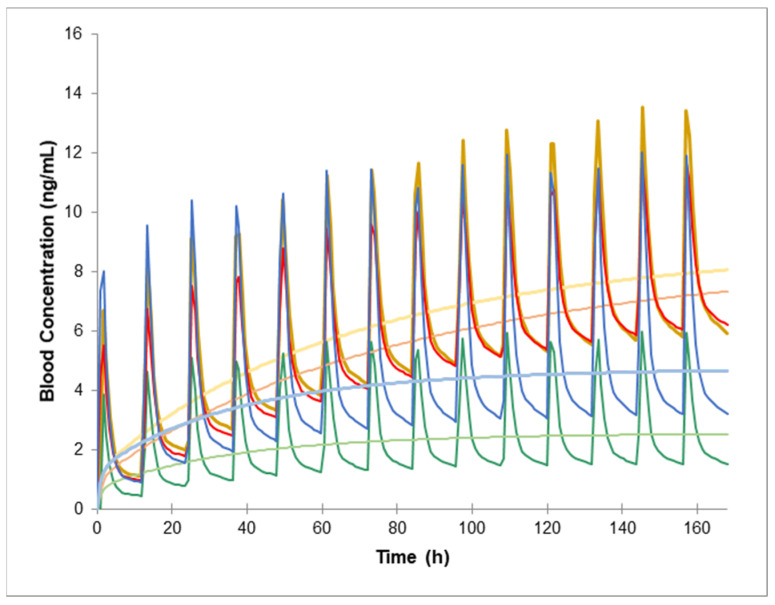
Predicted pharmacokinetic profiles of tacrolimus following intravenous (IV) infusion (light) and oral (PO) (bold) administration are illustrated for healthy volunteers (yellow), recipients only (red), donors only (blue), and both donor and recipient expressing CYP3A5 (*1/*1) (green).

When both donor and recipient are expressors of CYP3A5, the simulated AUCτ is significantly reduced, showing a nearly 70% decrease for the same PO dose. This reduction is less pronounced for the IV tacrolimus administration, leading to increased dose and Css/Cmin ratios (6.67 and 1.69, respectively) compared to the standard scenario (see Table 1 and Figure 2). When only the donor expresses CYP3A5, the AUCτ is somewhat less affected, though it is still reduced by 50%, relative to a population predominantly composed of CYP3A5 non-expressors (Table 1, Figure 2). Both the PO and IV administrations are similarly impacted in this case, resulting in a PO/IV dose ratio close to normal (4.0) but with an elevated Css/Cmin ratio (1.74). Conversely, when only the recipient expresses CYP3A5, a lower IV dose is necessary to achieve comparable oral exposure, leading to an increased PO/IV dose ratio (6.25) and a decreased Css/Cmin ratio (1.33) in comparison to the previous two scenarios.

### 2.4. Predicted Tacrolimus PO/IV Dose and Css/Cmin Ratios in Healthy Volunteers following PO and IV Administration of the CYP3A Inhibitor Itraconazole

**Figure 3:**
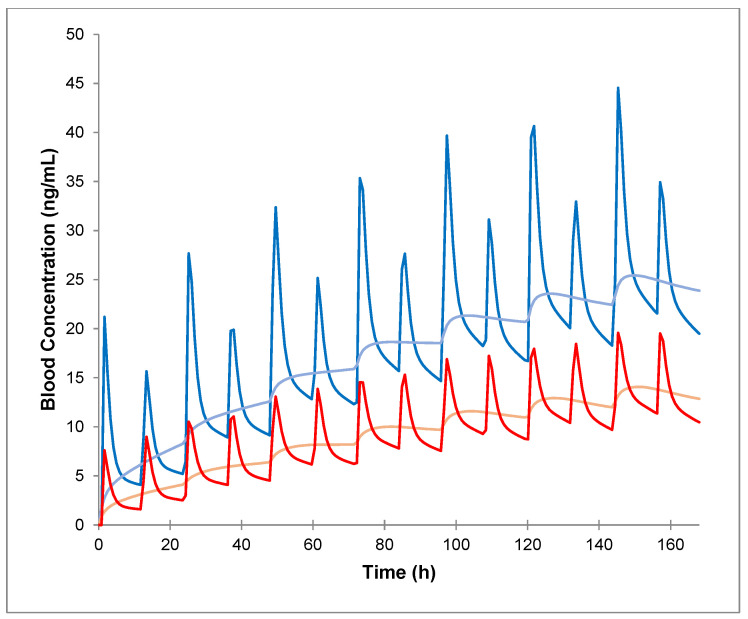
Predicted pharmacokinetic profiles of tacrolimus following intravenous (IV) infusion (light) and oral administration (bold) in healthy volunteers, in the presence of itraconazole administered PO (blue) and IV (red).

Simulated pharmacokinetic profiles following PO and IV administration of tacrolimus in healthy volunteers, in the presence of the CYP3A inhibitor itraconazole (administered both PO and IV) (Figure 3) demonstrated that blood exposure is more significantly affected for PO tacrolimus compared to IV tacrolimus. This effect resulted in a decrease in PO/IV dose ratio from 4.25 to 1.74 and a slight reduction in Css/Cmin ratio from 1.4 to 1.28 when itraconazole was PO-administered (Table 1). Conversely, when itraconazole was IV-administered, both the PO/IV dose ratios remained relatively unchanged, while the Css/Cmin ratio increased.

### 2.5. Comparison of PO and IV Tacrolimus: Predicted PO and IV Tacrolimus Doses and Css/Cmin Ratios Following PO and IV Administration of the CYP3A Inhibitor Itraconazole in Donors, Recipients, and in Both Donors and Recipients Expressing CYP3A5 (*1/*1)

**Figure 4:**
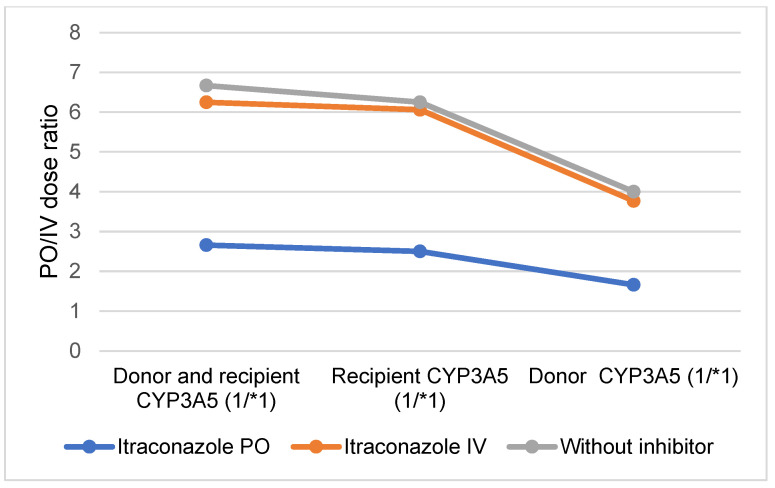
Predicted oral (PO) and intravenous (IV) tacrolimus dose ratios following PO and IV administration of the CYP3A inhibitor itraconazole in donors, recipients and both donors and recipients expressing CYP3A5 (*1/*1).

**Figure 5:**
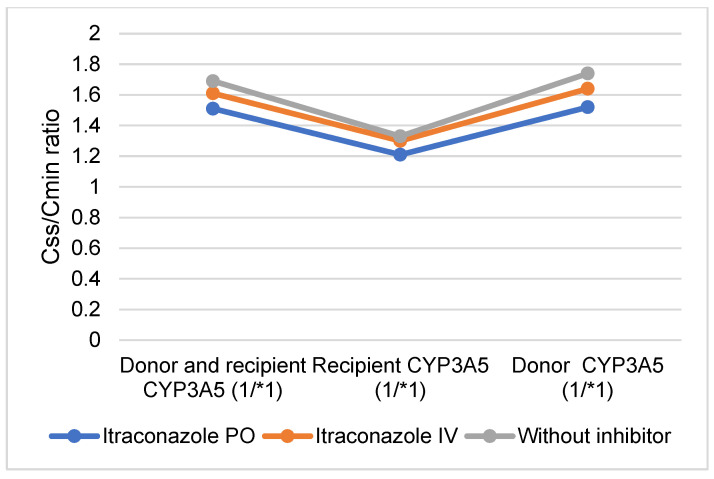
Predicted steady-state and trough concentrations (Css/Cmin) ratios following oral (PO) and intravenous (IV) administration of the CYP3A inhibitor itraconazole in donors, recipients and both donors and recipients expressing CYP3A5 (*1/*1).

When both the donor and recipient express functional CYP3A5, the reduction in tacrolimus blood exposure is somewhat mitigated by the PO administration of itraconazole (Figure 3 and Figure 4, Table S2). However, a higher IV dose of tacrolimus was required to maintain comparable exposure levels, resulting in a decreased PO/IV dose ratio and an increased Css/Cmin ratio (2.66 and 1.51, respectively). When only the recipient expresses CYP3A5 (*1/*1), the increase in exposure due to PO itraconazole administration was only minimally counteracted by the functional CYP3A5 in the intestine. Consequently, a higher IV dose of tacrolimus was needed to achieve the same blood exposure, leading to a PO/IV ratio similar to that observed in individuals with full CYP3A5 (*1/*1) expression (2.5) (Figure 4 and Figure 5, Table S2). For donors expressing CYP3A5, the increase in tacrolimus blood exposure was partially offset by the CYP3A5 expression in the liver. Despite this, tacrolimus metabolism remains elevated, necessitating a higher dose to maintain the same exposure as PO tacrolimus, which results in a decreased PO/IV dose ratio compared to a scenario with full CYP3A5 expression (1.66) (Figure 4 and Figure 5, Table S2).

When itraconazole was IV-administered, the reduction in tacrolimus blood exposure caused by the presence of functional CYP3A5 was largely unaffected by the inhibitor. Consequently, the ratios were comparable to those observed in the absence of the inhibitor across all scenarios of CYP3A5 expression (*1/*1) (Figure 3 and Figure 4, Table S2).

## 3. Discussion

This study estimated the Css/Cmin ratio during the switch between IV and PO administration of tacrolimus and successfully applied it to a clinical case without immediate clinical transplant-related complications. A 2005 prospective study [7] proposed a non-compartmental method of Css based on Cmin, in 15 post-renal transplant patients, which yielded achieving the same Css/Cmin ratio of 1.40 as derived from our PBPK simulations. Current guidelines rely on a PO/IV dose ratio based on achieving the same target for Css and Cmin, which may result in tacrolimus underexposure [18]. The International Society for Heart and Lung Transplantation (ISHLT) and another observational study recommend a PO/IV dose ratio of 5 [18,19], which is slightly higher than our actual estimate of 4.25. However, this lower PO/IV ratio aligns with the increased IV dose required to achieve similar blood exposure between the two routes of administration when Css is distinguished from Cmin.

For a highly bound, low extraction ratio drug such as tacrolimus, clearance can be influenced by changes in hematocrit and hepatic clearance [4,20]. Consequently, the Css/Cmin ratio was also simulated using a virtual twin strategy [16] to account for patient demographics, presumed CYP3A5 genotype, and hematocrit. This simulation resulted in a slight decrease in both the Css/Cmin and PO/IV ratios (1.25 vs. 1.40 and 3.92 vs. 4.25, respectively), primarily due to the presence of a small percentage of CYP3A5 (*1/*1) expressors in the healthy volunteer population. This presence reduced the simulated Cmin in the population simulation, thereby increasing the ratios compared to the clinical case [16]. An additional simulation incorporating decreased liver function, as observed early after liver transplantation, showed a further slight reduction in the ratios, which remained stable even with a significant decrease in liver function (90%). Thus, the above-mentioned ratio of 1.40 is mostly applicable to liver transplant patients with a functional graft, while a slight decrease in the Css/Cmin ratio might be expected, particularly in the early days following transplantation.

We utilized PBPK simulations to investigate various clinical scenarios and their effects on the Css/Cmin and PO/IV dose ratios. In the second scenario, where both donor and recipient express CYP3A5 (*1/*1), there is a dramatic 70% reduction in tacrolimus blood exposure following PO administration compared to a Caucasian population predominantly consisting of CYP3A5 non-expressors. This finding aligns closely with observed data in kidney transplant recipients [21]. When administered IV, the effect on tacrolimus blood exposure was less pronounced, likely due to reduced hepatic first-pass and intestinal drug extraction, a mechanism previously described in a case report on fluconazole [22]. Consequently, a higher dose of tacrolimus is required to achieve the same tacrolimus blood exposure as an IV dose. In this scenario, the target tacrolimus Css should be approximately 1.7 times the target Cmin, resulting in an increased PO/IV ratio, as observed in a retrospective study involving patients across various types of transplantation [23]. Interestingly, the AUCτ is less affected when only the recipient expresses functional CYP3A5. This observation aligns with a retrospective study indicating that the native intestinal CYP3A5 genotype was not associated with the occurrence of acute rejection, in contrast to the donor CYP3A5 genotype [24]. Several studies suggest that CYP3A4 expression significantly predominates over CYP3A5 expression in human small intestinal enterocytes, implying that CYP3A5 plays a minor role in the intestinal metabolism of CYP3A substrates in most individuals [25]. In the third scenario, where itraconazole was administered PO in a healthy population, a lower PO/IV ratio of 1.74 and a lower Css/Cmin ratio of 1.28 were observed, likely due to the inhibition of both intestinal and hepatic CYP3A by PO itraconazole [8]. A retrospective study in patients undergoing allogeneic hematopoietic stem cell transplantation also reported a similar decrease in the PO/IV ratio among patients treated with azoles [12]. When itraconazole was administered IV, its inhibitory effect was diminished, affecting IV and PO tacrolimus almost equally. This increase in tacrolimus exposure was consistent with observations in allogeneic hematopoietic stem cell transplant recipients, where dose reductions in tacrolimus ranged from 50 to 100% [13]. As the effect was similar for both routes of administration, the PO/IV and Css/Cmin ratios did not change significantly compared to the normal scenario. In the final and more complex scenario, the presence of functional CYP3A5 in both the donor and recipient was counteracted by PO itraconazole. A clinical study investigating this combination in solid organ transplant patients similarly showed that the increase in tacrolimus exposure due to azoles was completely blunted by the presence of functional CYP3A5 [26]. Since IV tacrolimus was less affected than PO tacrolimus, a higher dose was required to maintain comparable exposure, resulting in a decreased PO/IV dose ratio and an increased Css/Cmin ratio compared to the normal scenario. As previously mentioned, the impact of intestinal functional CYP3A5 on tacrolimus AUCτ was less pronounced when administered with PO itraconazole. In this context, the increase in tacrolimus exposure was only partially mitigated by the presence of functional CYP3A5, leading to a moderate increase in the tacrolimus IV dose. Since the IV tacrolimus was not affected by intestinal CYP3A5 but still subject to itraconazole inhibition, the PO/IV dose ratio remained largely unchanged compared to a scenario with full CYP3A5 expression in both donor and recipient. As observed earlier, IV itraconazole [27] had a reduced effect on tacrolimus AUCτ, with ratios approaching those seen in the absence of the inhibitor. This result confirms that ketoconazole has a significant inhibitory effect on intestinal CYP3A [28], which can markedly influence the intestinal absorption of a drug with low availability, such as tacrolimus [29].

Although the results align with ratios previously observed for tacrolimus in the literature, a primary limitation of this study is the lack of formal verification of the simulations with in vivo data. Additionally, most scenarios were based on a healthy human volunteer population. Consequently, the ratios presented are most applicable to patients with normal liver function and hematocrit levels, and decreased ratios might be expected in patients with reduced liver function and/or hematocrit, as seen early after transplantation [30]. Furthermore, as bioavailability can vary between different forms of PO tacrolimus administration such as granules or oral suspension, the present results may not be directly applicable to all oral formulations. Nonetheless, this study is the first to propose Css/Cmin ratios for tacrolimus across various complex clinical scenarios and can thus serve as a guide for clinicians when determining target tacrolimus blood concentrations during IV/PO switchover.

## 4. Materials and Methods

### PBPK Simulation

The absorption, distribution, metabolism, and excretion (ADME) simulator SimCYP^®^ version 21 software (Certara, SimCYP Ltd., Sheffield, UK) was used as the platform for the PBPK simulation. A tacrolimus compound file, which was previously validated in healthy volunteers and verified for drug–drug interactions by Hong et al. [12] was utilized for all simulations without further modification. The input parameters of the original model are detailed in Table S2. The model developed by Hong et al. did not incorporate P-glycoprotein (P-gp) transport, as its impact on tacrolimus absorption and hepatic elimination has been inconsistent [31,32,33]. Simulations were performed to match the AUCτ between twice-daily PO tacrolimus 1 mg and continuous IV infusion to estimate the bioavailability by dose ratio (PO/IV) and the Css/Cmin ratio following IV infusion and PO administration, respectively. The AUCτ was calculated using the log-trapezoidal rule, from 156 to 168 h, to ensure that tacrolimus had reached steady-state concentrations. The AUCτ, Css, and Cmin were reported in ng/mL·h and ng/mL, respectively. All PK parameters are presented as geometric means unless otherwise specified. All concentrations were simulated in blood.

The clinical case was simulated by creating a virtual twin of the patient. Age, sex, weight, height, hematocrit, and 3A genotype were matched to the patient and donor. A 3A5 (*3/*3) genotype was considered for the liver and other organs in the simulation, as both the patient and the donor were Caucasian. Liver function was considered normal since the graft was 1 month old, and regular follow-up of graft blood flow was normal [17]. Because the age limit for the healthy volunteer population is 65 years, the specific population models for Caucasian older adults, identified as ‘Sim-Geriatric NEC’ in the Simcyp population model library was used [34]. Ten trials of 1 participant each were simulated. To simulate a decrease in liver function, we successively decreased the CYP3A4 and CYP3A5 enzymatic abundance in the liver by 50, 75, and 90%. The liver flow and organ size were not modified because the liver transplant was considered successful. As a normal functional liver graft was considered, the rest of the scenarios were conducted using the Simcyp Caucasian population “Sim Healthy Volunteers”, and ten trials of 100 participants were simulated for each clinical scenario. In the “Sim Healthy Volunteers” population, 17% of the participants are CYP3A5 expressors.

The first scenario was conducted in a population where either the liver donor, the recipient, or both express CYP3A5 (*1/*1). To simulate a population of liver donors expressing CYP3A5 (*1/*1), the frequency of the population having extensive metabolism for CYP3A5 was set to 100% and enzyme abundances for CYP3A5 were set to 0 for both the intestine and colon, while the enzyme abundances in the liver were left unchanged. The opposite was performed for the population of recipients expressing CYP3A5 (*1/*1). The second scenario tested the administration of tacrolimus (IV or PO) in the presence and absence of the strong 3A/Pgp inhibitor itraconazole at a dose of 200 mg once daily (IV or PO). The Simcyp compound “itraconazole_Fasted Soln” was used with the addition of an inhibitory effect on CYP3A5. The compound had been successfully used to a simulate drug–drug interaction with a CYP3A4 substrate [35]. The input parameters can be found in Table S4. The third scenario explored a complex drug–drug interaction, conducted in donor, recipient, and both expressing CYP3A5 (*1/*1), with and without the CYP3A inhibitor itraconazole administered at a dose of 200 mg once daily (IV or PO).

## 5. Conclusions

Using PBPK approaches, the PO/IV dose ratios and Css/Cmin ratios were estimated to improve dose adjustment when considering IV/PO tacrolimus switching in transplant patients and to guide clinicians at the bedside. To our knowledge, this is the first time that Css/Cmin have been proposed in different clinical settings to avoid subtherapeutic tacrolimus blood exposure. Further research is encouraged to verify these estimates with in vivo data on an individualized basis.

## Figures and Tables

**Table 1:** Predicted oral/intravenous (PO/IV) doses and steady-state/trough concentrations (Css/Cmin) ratios after IV tacrolimus infusion and PO administration for similar simulated AUCτ in different clinical scenarios.

	Administration Route PO			Administration Route IV					
	Dose PO (mg/12 H)	AUCτ (ng/mL·h) (Geometric Mean)	Cmin (ng/mL)	Dose IV (mg/24 H)	AUCτ (ng/mL·h) (Geometric Mean)	Css (ng/mL)	Ratio AUCτ	Ratio PO/IV Dose	Ratio Css/Cmin
**Healthy volunteers**	**1**	95.29 [90%CI 81.84–110.95]	5.77 [90%CI 4.86–6.84]	**0.47**	95.91 [90%CI 87.59–105.02]	8.06 [90%CI 7.36–8.83]	**1.00**	**4.25**	**1.40**
**Clinical case (virtual twin)**	**1**	103.67 [90%CI 86.19–124.70]	7.09 [90%CI 5.83–8.62]	**0.51**	104.84 [90%CI 85.43–128.65]	8.85 [90%CI 6.82–10.21]	**0.99**	**3.92**	**1.25**
Both donor and recipient expressing CYP3A5 (*1/*1)	**1**	30.76 [90%CI 28.11–33.65]	1.50 [95%CI 1.34–1.67]	**0.30**	30.27 [90%CI 28.31–32.37]	2.53 [90%CI 2.36–2.70]	**1.00**	**6.67**	**1.69**
Donor expressing CYP3A5 (*1/*1)	**1**	50.52 [90%CI 45.41–56.21]	2.44 [90%CI 2.16–2.76]	**0.50**	50.80 [90%CI 47.16–54.73]	4.24 [90%CI 3.94–4.57]	**0.99**	**4.0**	**1.74**
Recipient expressing CYP3A5 (*1/*1)	**1**	78.55 [90%CI 71.71–86.03]	5.05 [90%CI 4.58–5.57]	**0.32**	79.78 [90%CI 74.45–85.49]	6.72 [90%CI 3.94–4.57]	**0.98**	**6.25**	**1.33**
**CYP3A inhibitor (itraconazole PO) healthy volunteers**	**1**	333.11 [90%CI 258.88–388.14]	22.62 [90%CI 19.16–26.71]	**1.15**	335.60 [90%CI 301.76–373.22]	29.03 [90%CI 26.11–32.27]	**0.99**	**1.74**	**1.28**
**CYP3A inhibitor (itraconazole IV) healthy volunteers**	**1**	158.85 [90%CI 134.31–187.88]	10.36 [90%CI 8.62–12.46]	**0.50**	159.62 [90%CI 143.20–178.58]	13.94 [90%CI 12.49–15.56]	**1.00**	**4.00**	**1.35**

## Data Availability

The simulation data are available upon request to interested researchers.

## Supplementary Materials

The following supporting information can be downloaded at: https://www.mdpi.com/article/10.3390/ph17081047/s1, Figure S1. General workflow of the study. PO = oral. IV = intravenous. Table S1. Predicted oral/intravenous (PO/IV) doses and steady-state/tough concentrations (Css/Cmin) ratios after tacrolimus IV infusion and PO administration for a 50%, 75%, and 90% reduction in CYP3A liver abundance for the clinical case. Table S2. Comparison between oral (PO) and intravenous (IV) Tacrolimus: Predicted PO and IV tacrolimus doses and steady-state/tough concentrations (Css/Cmin) ratios after PO and IV administration of the CYP3A inhibitor itraconazole in donors expressing CYP3A5 (*1/*1), recipients, and in both donors and recipients expressing CYP3A5 (*1/*1). Table S3. Parameters used in the PBPK model for tacrolimus, developed by Hong et al. for Simcyp^®^ Version 21 (Certara). Table S4. Parameters used in the PBPK model for Simcyp^®^ compound “itraconazole_Fasted Soln” Version 21 (Certara). References [36,37,38,39,40,41] are cited in the supplementary materials.
