# Supplementary material for: Guidance on Selecting Optimal Steady-State Tacrolimus Concentrations for Continuous IV Perfusion: Insights from Physiologically Based Pharmacokinetic Modeling
Source: Pharmaceuticals (Basel). 2024 Aug 8;17(8):1047. doi: 10.3390/ph17081047 (PMC11357179; doi:10.3390/ph17081047)
Supplement: Supplementary file 1 [file pharmaceuticals-17-01047-s001.zip › pharmaceuticals-3102899-supplementary.pdf]

Supplementary Online Content

Guidance on Selecting Optimal Steady-State Tacrolimus Concentrations for Continuous IV Perfusion: Insights from Physiologically Based Pharmacokinetic Modeling

Martischang Romain [1], Argyro Nikolaou [2], Youssef Daali [2,3,4], Caroline Flora Samer [2,3,4] and Jean Terrier [1,2,*]

[1] Division of General Internal Medicine, Geneva University Hospitals, 1205 Geneva, Switzerland
[2] Division of Clinical Pharmacology and Toxicology, Department of Anesthesiology, Pharmacology, Intensive Care and Emergency Medicine, Geneva University Hospitals, 1205 Geneva, Switzerland
[3] School of Pharmaceutical Sciences, Institute of Pharmaceutical Sciences of Western Switzerland, University of Geneva, 1205 Geneva, Switzerland
[4] Faculty of Medicine, University of Geneva, 1205 Geneva, Switzerland
* Correspondence: jean.terrier@hug.ch

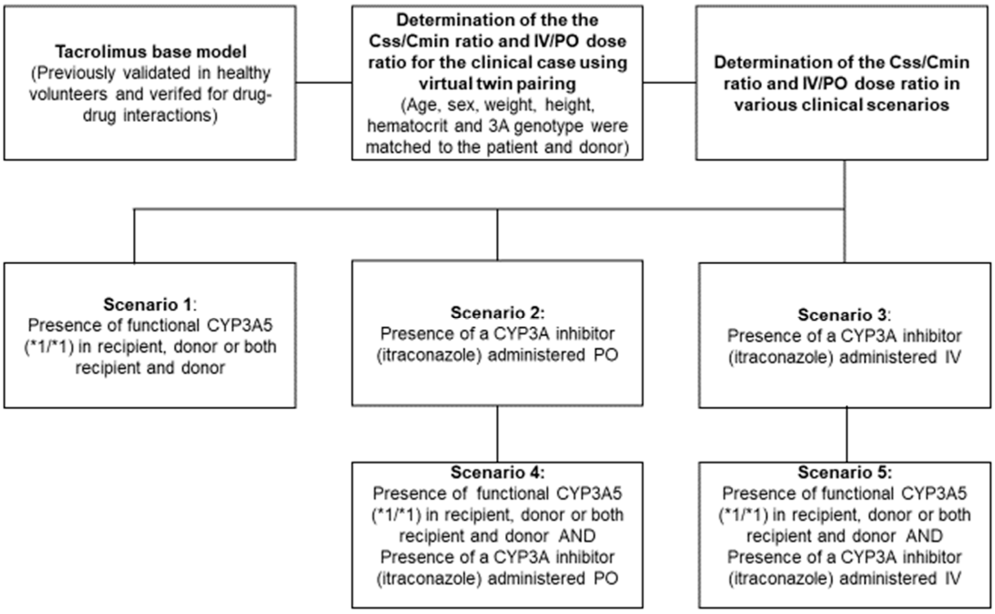

Figure S1. General workflow of the study. PO = oral. IV = intravenous

Table S1. Predicted oral/intravenous (PO/IV) doses and steady-state/tough concentrations (Css/Cmin) ratios after tacrolimus IV infusion and PO administration for a 50%, 75%, and 90% reduction in CYP3A liver abundance for the clinical case.

	**administration route PO**			**administration route IV**					
	**Dose PO (mg/12H)**	AUCτ (ng/ml*h) (geometric mean)	**Cmin (ng/ml)**	**Dose IV (mg/24H)**	AUC τ (ng/ml*h) (geometric mean)	**Css (ng/ml)**	**Ratio AUC τ**	**Ratio PO/IV dose**	**Ratio Css/Cmin**
**50% reduction in CYP3A liver abundance**	**1**	152.51 [90%CI 130.00 – 178.90]	10.89 [90%CI 9.26– 12.81]	**0.51**	150.02 [90%CI 123.97 – 181.56]	12.77 [90%CI 10.55 – 15.47]	**1.02**	**3.92**	**1.17**
**75% reduction in CYP3A liver abundance**	**1**	190.84 [90%CI 164.47 – 190.84]	13.87 [90%CI 11.95 – 16.10]	**0.52**	188.84 [90%CI 156.47 – 226.94]	16.13 [90%CI 13.40 – 19.43]	**1.01**	**3.84**	**1.16**
**90% reduction in CYP3A liver abundance**	**1**	222.70 [90%CI 192.58 – 257.53]	16.33 [90%CI 14.13 – 18.88]	**0.53**	221.74 [90%CI 184.18 – 266.96]	19.07 [90%CI 15.84 – 22.95]	**1**	**3.77**	**1.17**

Table S2. Comparison between oral (PO) and intravenous (IV) Tacrolimus: Predicted PO and IV tacrolimus doses and steady-state/tough concentrations (Css/Cmin) ratios after PO and IV administration of the CYP3A inhibitor itraconazole in donors expressing CYP3A5 (*1/*1), recipients, and in both donors and recipients expressing CYP3A5 (*1/*1).

	**administration route PO**			**administration route IV**					
	**Dose PO (mg/12H)**	AUCτ (ng/ml*h) (geometric mean)	**Cmin (ng/ml)**	**Dose IV (mg/24H)**	AUCτ (ng/ml*h) (geometric mean)	**Css (ng/ml)**	**Ratio AUCτ**	**Ratio PO/IV dose**	**Ratio Css/Cmin**
**Both donor and recipient expressing CYP3A5 (*1/*1) + Pgp/CYP3A inhibitor (itraconazole PO)**	**1**	101.06 [90%CI 89.84 – 113.68]	5.93 [90%CI 5.19– 6.78]	**0.75**	102.24 [90%CI 93.49 – 111.80]	8.97 [90%CI 8.20 – 9.81]	**0.99**	**2.66**	**1.51**
**Donor expressing CYP3A5 (*1/*1) + Pgp/CYP3A inhibitor (itraconazole PO)**	**1**	166.15 [90%CI 146.33 – 188.66]	9.55 [90%CI 8.28 – 11.03]	**1.2**	165.73 [90%CI 150.16 – 182.92]	14.54 [90%CI 13.17 – 16.05]	**1.00**	**1.66**	**1.52**
**Recipient expressing CYP3A5 (*1/*1) + Pgp/CYP3A inhibitor (itraconazole PO)**	**1**	288.16 [90%CI 259.86 – 319.55]	20.57 [90%CI 18.46 – 22.92]	**0.8**	287.83 [90%CI 264.83 – 312.84]	24.80 [90%CI 22.80 – 26.97]	**1.00**	**2.5**	**1.21**
**Both donor and recipient expressing CYP3A5 (*1/*1) + Pgp/CYP3A inhibitor (itraconazole IV)**	**1**	48.64 [90%CI 43.37 – 54.55]	2.64 [90%CI 2.31– 3.02]	**0.32**	47.66 [90%CI 43.27 – 52.50]	4.24 [90%CI 3.85 – 4.67]	**1.02**	**6.25**	**1.61**
**Donor expressing CYP3A5 (*1/*1) + Pgp/CYP3A inhibitor (itraconazole IV)**	**1**	80.60 [90%CI 70.48 – 92.18]	4.34 [90%CI 3.73 – 5.06]	**0.53**	79.86 [90%CI 71.77 – 88.86]	7.12 [90%CI 6.39 – 7.92]	**1.01**	**3.77**	**1.64**

**Recipient expressing CYP3A5 (*1/*1) + Pgp/CYP3A inhibitor (itraconazole IV)**	**1**	130.98 [90%CI 117.67 – 145.78]	8.98 [90%CI 8.02 – 10.04]	**0.33**	129.93 [90%CI 119.00 – 141.87]	11.27 [90%CI 10.32 – 12.32]	**1.01**	**6.06**	**1.30**

Table S3. Parameters used in the PBPK model for tacrolimus, developed by Hong et al. for Simcyp® Version 21 (Certara).

Parameter	Value	Source
**Physiochemical properties**		
**Molecular weight (g/mol)**	804.02	Drug label
**Log $P_{o:w}$**	3.3	Gertz et al. [1]
**Compound type**	Neutral	Gertz et al. [1]
**B/P**	35	Gertz et al. [1]
**fu_p**	0.013	Gertz et al. [1]
**Absorption**		
**Absorption model**	First-order model	
**Caco-2 permeability (10^{-6} cm/s)**	13.1	Gertz et al. [2]
**Scalar**	2.157	Gertz et al. [2]
**fu_{gut}**	1	Default
**k_a (h^{-1})**	3.68	Emoto et al. [3]
**f_a**	1.0	Emoto et al. [3]
**Lag time (h)**	0.43	Emoto et al. [3]
**Qgut (L/h)**	13.3	Predicted by Simcyp
**$P_{eff,man}$ (×10^{-4} cm/s)**	3.52	Predicted by Simcyp
**Distribution**		
**Distribution model**	Minimal PBPK model	
**k_{in} (h^{-1})**	0.68	Emoto et al. [3]
**k_{out} (h^{-1})**	0.10	Emoto et al. [3]
**V_{sac} (l/kg)**	10.8	Emoto et al. [3]
**V_{ss} (l/kg)**	18.0	Predicted by Simcyp Method 1
**Elimination**		
**13-*O*-desmethylation**		
**CYP3A4 Vmax (pmol/min/pmol CYP)**	8	Dai et al. [4]
**CYP3A4 Km (μM)**	0.21	Dai et al. [4]
**CYP3A5 Vmax (pmol/min/pmol CYP)**	17	Dai et al. [4]
**CYP3A5 Km (μM)**	0.21	Dai et al. [4]
**12-hydroxylation**		
**CYP3A4 Vmax (pmol/min/pmol CYP)**	0.6	Dai et al. [4]
**CYP3A4 Km (μM)**	0.29	Dai et al. [4]
**CYP3A5 Vmax (pmol/min/pmol CYP)**	1.4	Dai et al. [4]
**CYP3A5 Km (μM)**	0.35	Dai et al. [4]
**CYP3A4, 3A5 ISEF**	0.24	Simcyp default
**Renal clearance (L/h)**	0	Moller et al. [5]

B/P, blood-to-plasma ratio; CL_{int}, intrinsic clearance; CL_{po}, in vivo oral clearance; f_a, fraction available from dosage form; fu_{gut}, fraction unbound in the enterocyte; fu_p, fraction unbound in plasma; ISEF, inter-system extrapolation factor; k_a, absorption rate constant; k_{in} and k_{out}, first-order rate constants describing the drug transfer to a single adjusting compartment; Km, Michaelis constant; Log $P_{o:w}$, logarithmic partition coefficient octonal:water; $P_{eff,man}$, effective permeability in man; pK_a, logarithm of acid dissociation constant; Q, inter-compartment clearance; Qgut, flow rate for overall delivery of drug to the gut; Vmax, maximum metabolic rate; V_{sac}, single adjusted compartment volume; V_{ss}, volume of distribution at steady state.

Table S4. Parameters used in the PBPK model for Simcyp® compound "itraconazole_Fasted Soln" Version 21 (Certara).

Parameter	Value	Source
**Physiochemical properties**		
**Molecular weight (g/mol)**	705.6	Simcyp® Default setting
**Log $P_{o:w}$**	4.47	Simcyp® Default setting
**Compound type**	Monoprotic base	Simcyp® Default setting
**pKa 1**	4.28	Simcyp® Default setting
**B/P**	0.58	Simcyp® Default setting
**fu_p**	0.016	Simcyp® Default setting
**Absorption**		
**Absorption model**	First-order model	
**fu_{gut}**	0.016	Simcyp® Default setting
**k_a (h^{-1})**	1.5	Simcyp® Default setting
**f_a**	1.0	Simcyp® Default setting
**Lag time (h)**	0	Simcyp® Default setting
**Qgut (L/h) (CV%)**	18.32 (12)	Simcyp® Default setting
**Distribution**		
**Distribution model**	Minimal PBPK model	
**k_{in} (h^{-1})**	0	Simcyp® Default setting
**k_{out} (h^{-1})**	0	Simcyp® Default setting
**V_{sac} (l/kg)**	0.00001	Simcyp® Default setting
**V_{ss} (l/kg)**	2.51	Predicted by Simcyp Method 1
**Elimination**		
**CYP1A2 Clint (uL/min/pmol of isoform)**	1	
**CYP3A4 Vmax (pmol/min/pmol CYP)**	0.065	Simcyp® Default setting
**CYP3A4 Km (µM)**	0.0039	Simcyp® Default setting
**CYP3A4 fumic**	1	Simcyp® Default setting
**Interaction**		
**CYP 3A4 Ki**	0.0013	Simcyp® Default setting
**CYP 3A5 Ki**	0.01	[6]

B/P, blood-to-plasma ratio; CL_{int}, intrinsic clearance; CL_{po}, in vivo oral clearance; f_a, fraction available from dosage form; fu_{gut}, fraction unbound in the enterocyte; fu_p, fraction unbound in plasma; ISEF, inter-system extrapolation factor; k_a, absorption rate constant; k_{in} and k_{out}, first-order rate constants describing the drug transfer to a single adjusting compartment; Km, Michaelis constant; Log $P_{o:w}$, logarithmic partition coefficient octonal:water; $P_{eff,man}$, effective permeability in man; pK_a, logarithm of acid dissociation constant; Q, inter-compartment clearance; Qgut, flow rate for overall delivery of drug to the gut; Vmax, maximum metabolic rate; V_{sac}, single adjusted compartment volume; V_{ss}, volume of distribution at steady state.

References

1. M. Gertz, J. B. Houston, et A. Galetin, « Physiologically based pharmacokinetic modeling of intestinal first-pass metabolism of CYP3A substrates with high intestinal extraction », *Drug Metab Dispos*, vol. 39, nº 9, p. 1633-1642, sept. 2011, doi: 10.1124/dmd.111.039248.
2. M. Gertz, A. Harrison, J. B. Houston, et A. Galetin, « Prediction of human intestinal first-pass metabolism of 25 CYP3A substrates from in vitro clearance and permeability data », *Drug Metab Dispos*, vol. 38, nº 7, p. 1147-1158, juill. 2010, doi: 10.1124/dmd.110.032649.
3. C. Emoto *et al.*, « A Theoretical Physiologically-Based Pharmacokinetic Approach to Ascertain Covariates Explaining the Large Interpatient Variability in Tacrolimus Disposition », *CPT Pharmacometrics Syst Pharmacol*, vol. 8, nº 5, p. 273-284, mai 2019, doi: 10.1002/psp4.12392.
4. Y. Dai *et al.*, « Effect of CYP3A5 polymorphism on tacrolimus metabolic clearance in vitro », *Drug Metab Dispos*, vol. 34, nº 5, p. 836-847, mai 2006, doi: 10.1124/dmd.105.008680.
5. A. Möller *et al.*, « The disposition of 14C-labeled tacrolimus after intravenous and oral administration in healthy human subjects », *Drug Metab Dispos*, vol. 27, nº 6, p. 633-636, juin 1999.
6. Y. Shirasaka *et al.*, « Effect of CYP3A5 expression on the inhibition of CYP3A-catalyzed drug metabolism: impact on modeling CYP3A-mediated drug-drug interactions », *Drug Metab Dispos*, vol. 41, nº 8, p. 1566-1574, août 2013, doi: 10.1124/dmd.112.049940.
